# Lifestyle, course of COVID-19, and risk of Long-COVID in non-hospitalized patients

**DOI:** 10.3389/fmed.2022.1036556

**Published:** 2022-10-24

**Authors:** Magdalena Pływaczewska-Jakubowska, Michał Chudzik, Mateusz Babicki, Joanna Kapusta, Piotr Jankowski

**Affiliations:** ^1^Department of Internal Medicine and Geriatric Cardiology, Centre of Postgraduate Medical Education, Warsaw, Poland; ^2^Department of Family Medicine, Wroclaw Medical University, Wroclaw, Poland; ^3^Department of Internal Medicine and Cardiac Rehabilitation, Medical University of Lodz, Łódz, Poland

**Keywords:** COVID-19, Long COVID-19, lifestyle, risk factors, SARS-CoV-2

## Abstract

**Introduction:**

The coronavirus disease (COVID) 2019 pandemic remains a great challenge for the healthcare system. The widely reported prolonged signs and symptoms resulting from severe acute respiratory syndrome coronavirus 2 (SARS-CoV-2) infection (Long-COVID) require medical care. The aim of the study was to assess factors, including lifestyle variables, related to the course of COVID-19 infection and to assess their impact on prolonged symptoms in non-hospitalized patients with COVID-19.

**Methods:**

A total of 1,847 (637 men and 1,210 women) non-hospitalized participants of the STOP-COVID registry of the PoLoCOV-Study who, following the COVID-19, underwent check-up examinations at the cardiology outpatient clinic were included in the analysis.

**Results:**

The study participants (median age 51 [41–62] years) were evaluated at 13.4 (8.4–23.6) weeks following the diagnosis of COVID-19. Female sex (odds ratio [OR] 1.46 [95% CI 1.19–1.78]), body mass index (BMI; per 1 kg/m^2^: 1.02 [1.00–1.04]), hypertension (1.39 [1.07–1.81]), asthma (1.55 [1.06–2.27]), stress or overworking (1.54 [1.25–1.90]), and nightshift work (1.51 [1.06–2.14]) were independently related to the severity of symptoms during acute phase of the COVID-19 infection. The Long-COVID syndrome was independently related to the female sex (1.42 [1.13–1.79]), history of myocardial infarction (2.57 [1.04–6.32]), asthma (1.56 [1.01–2.41]), and severe course of the acute phase of the COVID-19 infection (2.27 [1.82–2.83]).

**Conclusion:**

Female sex, BMI, asthma, hypertension, nightshifts, and stress or overworking are significantly related to the severity of the acute phase of the COVID-19 infection, while female sex, asthma, history of myocardial infarction, and the severity of symptoms in the acute phase of COVID-19 are the predictors of Long-COVID in non-hospitalized patients. We did not find an independent relation between Long-COVID and the studied lifestyle factors.

## Introduction

The global coronavirus disease 2019 (COVID-19) pandemic remains a great challenge for the healthcare systems, despite the fact that the case fatality rate is decreasing (1). The vast majority of patients following a severe acute respiratory syndrome coronavirus 2 (SARS-CoV-2) infection (Long-COVID) reported prolonged symptoms (2, 3). The disease is still not well understood. The Long-COVID syndrome is defined by the National Institute for Health and Care Excellence as “signs and symptoms that develop during or after an infection consistent with COVID-19 which continue for more than 12 weeks and are not explained by an alternative diagnosis” (4). The severity of the COVID-19 course is the most important variable for persistent signs and symptoms in the post-discharge period (5, 6). However, 60–80% of patients have a mild or asymptomatic SARS-CoV-2 infection (7, 8). Some individuals are more prone to have a severe course or even develop respiratory failure quickly (9). These studies have shown that comorbidities, especially cardiovascular and pulmonary diseases, are associated with a risk of hospitalization and worse outcomes (10, 11). Furthermore, not only comorbidities influence COVID-19. A growing number of scientific reports concern lifestyle variables as the severity of SARS-CoV-2 infection, such as improper eating habits and a lack of physical activity (10, 12, 13).

Most of the in-depth analyses focused on hospitalized patients who were accurately diagnosed and obtained appropriate treatment and rehabilitation during the acute phase of illness, even though the majority of individuals infected with SARS-CoV-2 were patients isolated and treated at home. However, the epidemiological data indicate that the vast majority of COVID-19 patients are treated at home (8). The factors related to the course of the COVID-19 disease and the development of the Long-COVID syndrome in this population are not well understood. Therefore, the aim of this study was to determine factors, including lifestyle variables, related to the course of SARS-CoV-2 infection and to determine their impact on prolonged symptoms in non-hospitalized patients with COVID-19.

## Materials and methods

We analyzed the data of participants of the STOP-COVID registry of the PoLoCOV-Study (ClinicalTrials.gov identifier: NCT05018052) who, following COVID-19, underwent check-up examinations at an outpatient cardiac clinic and were at least 18 years of age. The patient follow-up period spanned from May 2020 to January 2022. The SARS-CoV-2 infection was confirmed by the RT-PCR test or antigen test in each study participant. We excluded patients who were hospitalized for COVID-19. Using standardized data collection forms, demographic and clinical details were collected, including the course of the disease, post-COVID-19 complaints, comorbidities, and lifestyle.

The subjective level of the COVID-19 symptoms was evaluated using a three-point scale: each patient was asked to indicate the severity of symptoms, with 1 point indicating no severe symptoms, 2 points indicating severe symptoms, and 3 points indicating very severe symptoms. Combining this scale with the duration of symptoms, maximal body temperature, dyspnoea, and blood-oxygen saturation, the severity of the COVID-19 infection was assessed. We defined asymptomatic or mild course as no symptoms or symptoms lasting up to 7 days and ranked by a participant as “1,” without temperature >38°C; moderate course as symptoms ranked as “2” or “3” with fever >38°C or dyspnoea lasting 1–3 days or symptoms of any severity lasting 7–14 days; and severe course as symptoms lasting more than 14 days or oxygen saturation below 94 with fever 38°C or dyspnoea lasting more than 3 days.

We analyzed the following lifestyle factors: physical activity (regular physical activity was defined as at least 150–300 min per week of moderate-intensity activity or 75–150 min per week of high-intensity activity during at least 3 months preceding COVID-19), stress, and overworking (anxious, on edge, not being able to stop or control worrying more than half a day) during 4 weeks preceding COVID-19, insomnia (defined as a difficulty falling asleep and maintaining sleep continuity during 4 weeks before COVID-19; falling asleep after midnight and nightshift work), and smoking (defined as using any tobacco products within the last 12 months).

Long-COVID-19 was defined as new or ongoing signs or symptoms associated with a SARS-CoV-2 infection persisting for more than 12 weeks (4).

The study was carried out in conformance with the Declaration of Helsinki and was approved by the Bioethics Committee of Lodz Regional Medical Chamber No. 0115/2021. All patients gave their informed consent.

### Statistical analysis

Continuous variables are presented as medians with first and third quartiles, while categorical values are presented as proportions with 95% confidence intervals (CIs), when appropriate. The Shapiro–Wilk test was used to assess the normality. Continuous variables were compared using the Mann–Whitney *U* test or the Kruskal–Wallis test. The Fisher's exact test or the Pearson χ^2^ test was applied to all categorical variables, when appropriate. Multivariate logistic analysis was used to assess factors independently related to the severity of the COVID-19 infection and the Long-COVID-19. A *P* < 0.05 was considered statistically significant. The statistics were calculated using the STATISTICA 13 software (TIBCO Software, Palo Alto, CA, USA).

## Results

A total of 1,847 (637 men and 1,210 women) patients (median age 51 [41–62] years; range: 16–85 years) were analyzed. Patients were evaluated at 13.4 [8.4–23.6] weeks following the diagnosis of COVID-19. Overall, 796 (43.1%) patients had an asymptomatic or mild course of the acute phase of the COVID-19 infection, 571 (30.9%) had a moderate course, and 480 (26.0%) suffered from a severe course. Patients with the asymptomatic or mild course of the acute phase of COVID-19 were younger compared to participants with more severe symptoms (Table 1). The proportion of women was higher among patients with mild, moderate, as well as severe courses of the COVID-19 disease, although the difference was significantly higher in groups with more severe courses of the disease. Overall, 1,148 (62.2%) patients had at least one comorbidity. The most common comorbidity was hypertension (33.4%), while 532 (28.8%) patients were obese. The results of the multivariate analysis are presented in Table 2. Sex, body mass index (BMI), hypertension, asthma, stress/overworking, going to bed after midnight, and nightshift work were the predictors of the severity of symptoms in the acute phase of COVID-19 after multivariable adjustments.

**Table 1:** The study group characteristics with regard to the course of the acute phase of the COVID-19 infection.

	**Course of COVID-19**			***p*-value**
	**Asymptomatic or mild**	**Moderate**	**Severe**	
	***N* = 796**	***N* = 571**	***N* = 480**	
Age [years]	50 [40–62]	52 [42–61]	52 [43–62]	0.019
Sex				0.002
Males	312, 39.2%	178, 31.2%	147, 30.6%	
Females	484, 60.8%	393, 68.8%	333, 69.4%	
BMI [kg/m^2^]	26.5 [23.3–30.4]	26.7 [23.8–31]	27.6 [24.3–31]	0.015
Post-COVID-19 signs and symptoms lasting ≤ 3 months	682	554	469	<0.001
	85.7%	97.0%	97.7%	
Post-COVID-19 signs and symptoms lasting >3 months	357	341	315	<0.001
	55.9%	70.8%	79.6%	
Any comorbidity	453	374	321	<0.001
	56.9%	65.5%	66.9%	
Hypertension	259	181	177	0.16
	32.5%	31.7%	36.8%	
Diabetes	66	54	45	0.7
	8.3%	9.5%	9.4%	
Dyslipidemia	140	122	98	0.18
	17.6%	21.4%	20.4%	
Coronary artery disease	33	24	28	0.32
	4.2%	4.2%	5.8%	
Myocardial infarction in the history	22	9	11	0.34
	2.8%	1.6%	2.3%	
Venous thromboembolism	11	7	1	0.22
	1.4%	1.2%	0.21%	
COPD	14	7	14	0.12
	1.8%	1.2%	2.9%	
Asthma	49	46	58	<0.001
	6.2%	8.1%	12.1%	
Life style				
Stress/overworking during	224	194	188	0.001
	29.0%	35.7%	40.6%	
Insomnia	145	124	112	0.067
	18.2%	21.7%	23.3%	
Falling asleep after midnight	95	67	77	0.063
	12.3%	12.3%	16.6%	
Nightshifts	60	52	52	0.11
	7.8%	9.6%	11.2%	
Insomnia or falling asleep after midnight or nightshifts	245	198	179	0.048
	30.8%	34.7%	37.3%	
Smoking	76	53	34	0.29
	9.6%	9.3%	7.1%	
Regular physical activity	231	131	128	0.07
	29.9%	24.1%	27.7%	

BMI, body mass index; COPD, chronic obstructive pulmonary disease.

**Table 2:** Multivariate analysis of predictors for a moderate or severe COVID-19 clinical course.

**Variable**		**Multivariable**		
	**Univariable**	**Model 1**	**Model 2**	**Model 3**
Age per 10 years	1.10 (1.03–1.18)	1.10 (1.02–1.17)	1.07 (1.02–1.17)	1.03 (0.96–1.12)
	*P =* 0.007	*P =* 0.01*	*P =* 0.58	*P =* 0.32
Females	1.42 (1.17–1.72)	1.41 (1.16–1.72)	1.47 (1.20–1.80)	1.46 (1.19–1.78)
	*P <* 0.001	*P <* 0.001**	*P <* 0.001	*P <* 0.001
BMI per 1 kg/m^2^	1.03 (1.01–1.04)	1.03 (1.01–1.05)	–	1.02 (1.00–1.04)
	*P =* 0.004	*P =* 0.003		*P =* 0.02
Any comorbidity	1.48 (1.22–1.79)	1.39 (1.13–1.71)	1.29 (1.04–1.61)	–
	*P <* 0.001	*P =* 0.002	*P =* 0.02	
Hypertension	1.07 (0.88–1.30)	0.97 (0.78–1.21)	0.87 (0.67–1.09)	1.39 (1.07–1.81)
	*P =* 0.49	*P =* 0.81	*P =* 0.22	*P =* 0.01
Diabetes	1.15 (0.83–1.59)	1.08 (0.77–1.53)	1.02 (0.71–1.45)	1.04 (0.72–1.5)
	*P =* 0.40	*P =* 0.65	*P =* 0.93	*P =* 0.82
Dyslipidemia	1.24 (0.98–1.57)	1.18 (0.93–1.51)	1.16 (0.90–1.49)	1.06 (0.81–1.38)
	*P =* 0.07	*P =* 0.17	*P =* 0.24	*P =* 0.68
Coronary artery disease	1.20 (0.77–1.88)	1.16 (0.73–1.85)	1.22 (0.75–1.97)	1.15 (0.71–1.86)
	*P =* 0.42	*P =* 0.54	*P =* 0.43	*P =* 0.57
Myocardial infarction in the history	1.47 (0.79–2.71)	1.40 (0.75–2.65)	1.40 (0.74–2.63)	1.49 (0.79–2.82)
	*P =* 0.22	*P =* 0.29	*P =* 0.30	*P =* 0.22
COPD	1.14 (0.57–2.26)	1.10 (0.54–2.23)	1.07 (0.52–2.17)	(0.53–1.92)
	*P =* 0.71	*P =* 0.79	*P =* 0.86	*P =* 0.98
Asthma	1.67 (1.18–2.38)	1.59 (1.11–2.28)	1.68 (1.16–2.43)	1.55 (1.06–2.27)
	*P =* 0.004	*P =* 0.01	*P =* 0.006	*P =* 0.023
Stress/overworking	1.50 (1.23–1.83)	1.57 (1.28–1.94)	1.51 (1,22–1.87)	1.54 (1.25–1.90)
	*P <* 0.001	*P <* 0.001	*P <* 0.001	*P <* 0.001
Insomnia	1.30 (1.03–1.64)	1.22 (0.96–1.54)	1.20 (0.95–1.52)	1.18 (0.93–1.49)
	*P =* 0.026	*P =* 0.09	*P =* 0.12	*P =* 0.17
Falling asleep after midnight	1.19 (0.90–1.57)	1.21 (0.91–1.60)	1.19 (0.89–1.59)	1.18 (0.88–1.57)
	*P =* 0.22	*P =* 0.19	*P =* 0.23	*P*=0.27
Nightshifts	1.37 (0.98–1.91)	1.52 (1.08–2.15)	1.48 (1.04–2.10)	1.51 (1.06–2.14)
	*P =* 0.063	*P =* 0.017	*P =* 0.029	*P =* 0.022
Insomnia or falling asleep after midnight or nightshifts	1.26 (1.03–1.53)	1.22 (1.0–1.49)	1.22 (1.00–1.49)	1.21 (0.99–1.47)
	*P =* 0.022	*P =* 0.047	*P =* 0.044	*P =* 0.063
Smoking	1.17 (0.85–1.61)	1.13 (0.81–1.57)	1.08 (0.77–1.51)	1.08 (0.77–1.52)
	*P =* 0.34	*P =* 0.47	*P =* 0.67	*P =* 0.64
Regular physical activity	0.81 (0.66–1.00)	0.85 (0.69–1.06)	0.89 (0.71–1.10)	0.89 (0.72–1.11)
	*P =* 0.053	*P =* 0.14	*P =* 0.28	*P =* 0.30

BMI, Body mass index; COPD, chronic obstructive pulmonary disease.

*Adjusted for sex.

**Adjusted for age.

Model 1 – Adjusted for age and sex.

Model 2 – Adjusted for age, sex, and body mass index.

Model 3 – Adjusted for age, sex, body mass index, and comorbidities.

In total, 92% of patients reported symptoms within the first 3 months following the COVID-19 infection. Overall, 1,517 patients were interviewed at least 3 months following the COVID-19 infection. Among them, 1,013 (66.8%) patients fulfilled the criteria for the Long-COVID syndrome (symptoms lasting over 12 weeks). Patients suffering from Long-COVID syndrome significantly more often were women and more often reported the presence of asthma (Table 3). The Long-COVID syndrome was significantly more often found in participants with severe course of the acute phase of COVID-19 (*n* = 315; 79.5%), compared to those with moderate (*n* = 341; 70.8%) and mild (*n* = 357; 56.9%) course of COVID-19 (*P* < 0.001). The results of the multivariate analysis are presented in Table 4. Sex, asthma, history of myocardial infarction, and the severity of symptoms in the acute phase of COVID-19 occurred to be significantly related to the Long-COVID after multivariable adjustments.

**Table 3:** Comparison of patients with and without the Long-COVID syndrome.

	**No Long-COVID syndrome**	**Long-COVID** **syndrome**	***p*-value**
	***N* = 504**	***N* = 1,013**	
Age [years]	51 [41–61]	52 [42–62]	0.22
Sex			0.003
Males	200 (39.7%)	324 (32.0%)	
Females	304 (60.3%)	689 (68.0%)	
BMI [kg/m^2^]	26.6 [23.5–30.5]	27.1 [24.1–30.9]	0.15
Any comorbidity	304	633	0.41
	60.3%	62.5%	
Hypertension	173	340	0.77
	34.3%	33.6%	
Diabetes	52	87	0.27
	10.3%	8.6%	
Dyslipidemia	86	212	0.07
	17.1%	20.9%	
Coronary artery disease	17	57	0.055
	3.4%	5.6%	
Myocardial infarction in the history	6	28	0.051
	1.2%	2.8%	
Venous thromboembolism in the history	5	14	0.52
	1.0%	1.4%	
COPD	9	23	0.54
	1.8%	2.3%	
Asthma	31	96	0.028
	6.2%	9.5%	
Lifestyle			
Stress/overworking	159	337	0.47
	31.6%	33.3%	
Insomnia	93	218	0.16
	18.5%	21.5%	
Falling asleep after midnight	62	135	0.56
	12.3%	13.3%	
Nightshifts	49	80	0.24
	9.7%	7.9%	
Insomnia or falling asleep after midnight or nightshifts	162	340	0.58
	32.1%	33.6%	
Smoking	43	91	0.77
	8.5%	9.0%	
Regular physical activity	266	123	0.42
	52.8%	12.1%	
Severe course of the acute COVID-19 phase	222	656	<0.001
	44.1%	64.8%	

BMI, body mass index; COPD, chronic obstructive pulmonary disease.

**Table 4:** Multivariate analysis of predictors of Long-COVID signs and symptoms.

**Variable**	**Univariable**	**Multivariable**		
		**Model 1**	**Model 2**	**Model 3**
Age per 10 years	1.05 (0.97–1.14)	1.05 (0.97–1.14)	1.04 (0.95–1.13)	1.04 (0.95–1.14)
	*P* = 0.26	*P* = 0.38*	*P* = 0.38	*P* = 0.40
Females	1.39 (1.12–1.74)	1.39 (1.11–.31)	1.42 (1.13–1.79)	1.42 (1.13–1.79)
	*P* = 0.003	*P* = 0.004**	*P* = 0.003	*P* = 0.003
BMI per 1 kg/m^2^	1.01 (0.99–1.03)	1.02 (0.99–1.04)	–	1.02 (0.99–1.04)
	*P* = 0.24	*P* = 0.15		*P* = 0.16
Any comorbidity	1.10 (0.88–1.37)	1.05 (0.83–1.34)	0.99 (0.77–1.28)	–
	*P* = 0.42	*P* = 0.68	*P* = 0.95	
Hypertension	1.03 (0.82–1.31)	1.08 (0.84–1.38)	1.13 (0.88–1.46)	1.17 (0.88–1.56)
	*P* = 0.78	*P* = 0.56	*P* = 0.34	*P* = 0.28
Diabetes	1.22 (0.85–1.76)	1.28 (0.88–1.86)	1.36 (0.92–1.99)	1.36 (0.93–2.01)
	*P* = 0.27	*P* = 0.2	*P* = 0.12	*P* = 0.12
Dyslipidemia	1.29 (0.98–1.70)	1.28 (0.96–1.70)	1.26 (0.95–1.68)	1.30 (0.96–1.76)
	*P* = 0.075	*P* = 0.091	*P* = 0.11	*P* = 0.088
Coronary artery disease	1.71 (0.98–2.97)	1.74 (0.99–3.08)	1.76 (0.99–3.12)	1.77 (0.99–3.14)
	*P* = 0.058	*P* = 0.056	*P* = 0.053	*P* = 0.052
Myocardial infarction in the history	2.36 (0.97–5.74)	2.51 (1.02–6.16)	2.55 (1.04–6.28)	2.57 (1.04–6.32)
	*P* = 0.058	*P* = 0.045	*P* = 0.038	*P* = 0.04
COPD	1.28 (0.59–2.78)	1.19 (0.54–2.62)	1.18 (0.54–2.60)	1.18 (0.54–2.60)
	*P* = 0.54	*P* = 0.67	*P* = 0.68	*P* = 0.69
Asthma	1.60 (1.05–2.43)	1.54 (1.01–2.35)	1.53 (1.00–2.33)	1.56 (1.01–2.41)
	*P* = 0.029	*P* = 0.045	*P* = 0.049	*P* = 0.043
Stress/overworking during 4 weeks preceding COVID-19	1.09 (0.86–1.37)	1.10 (0.87–1.40)	1.10 (0.87–1.40)	1.10 (0.87–1.40)
	*P* = 0.48	*P* = 0.41	*P* = 0.41	*P* = 0.41
Insomnia	1.21 (0.92–1.59)	1.15 (0.88–1.52)	1.15 (0.87–1.51)	1.15 (0.87–1.51)
	*P* = 0.16	*P* = 0.30	*P* = 0.32	*P* = 0.32
Falling aslee*P* after midnight	1.10 (0.80–1.52)	1.13 (0.82–1.57)	1.17 (0.84–1.63)	1.17 (0.84–1.63)
	*P* = 0.56	*P* = 0.45	*P* = 0.35	*P* = 0.35
Nightshifts	1.25 (0.86–1.82)	1.18 (0.78–1.77)	1.19 (0.81–1.74)	1.19 (0.81–1.74)
	*P* = 0.24	*P* = 0.43	*P* = 0.38	*P* = 0.38
Insomnia or falling aslee*P* after midnight or nightshifts	1.07 (0.85–1.34)	1.06 (0.84–1.33)	1.08 (0.86–1.36)	1.08 (0.86–1.37)
	*P* = 0.58	*P* = 0.62	*P* = 0.51	*P* = 0.51
Smoking	1.06 (0.72–1.55)	1.10 (0.75–1.62)	1.07 (0.73–1.59)	1.08 (0.73–1.59)
	*P* = 0.77	*P* = 0.61	*P* = 0.72	*P* = 0.72
Regular physical activity	0.90 (0.70–1.16)	0.86 (0.67–1.11)	0.86 (0.67–1.11)	0.86 (0.66–1.11)
	*P* = 0.42	*P* = 0.25	*P* = 0.24	*P* = 0.24
Severe course of the acute phase of the infection	2.33 (1.88–2.90)	2.28 (1.83–2.84)	2.27 (1.82–2.82)	2.27 (1.82–2.83)
	*P* <0.001	*P* <0.001	*P* <0.001	*P* <0.001

BMI, Body mass index; COPD, chronic obstructive pulmonary disease.

*Adjusted for sex.

**Adjusted for age.

Model 1 – Adjusted for age and sex.

Model 2 – Adjusted for age, sex, and body mass index.

Model 3 – Adjusted for age, sex, body mass index, and comorbidities.

## Discussion

Long-COVID disease is still not well understood. The risk factors, course, and treatment of the disease are still not clear. In addition, most of the data are based on patients who have been hospitalized with COVID-19. Therefore, the purpose of this study was to evaluate risk factors, including selected aspects of lifestyle, chronic conditions, and the course of COVID-19, on the risk of developing Long-COVID.

According to research data, a dysregulated immune response, immunothrombosis, endothelial dysfunction, and multiple organ damage have an impact on the occurrence of Long-COVID-19 syndrome (14, 15). Our study demonstrated that the severity of signs and symptoms during the acute phase of infection is the strongest risk factor for Long-COVID-19. Sudre et al. reported that out of more than 4,000 patients who suffered from COVID-19, Long-COVID-19 occurred three times more often in individuals who had more than 5 signs and symptoms during the first week of SARS-CoV-2 infection (odds ratio [OR] 3.53, 95% CI 2.76–4.50) (14). In the PHOSP-COVID study, only 29% of 1,077 patients discharged following COVID-19, felt fully recovered during the second to the seventh month. Female sex, middle age (40–59 years), two or more comorbidities, and more severe signs and symptoms during acute illness were the factors associated with a non-recovery (16). Augustin et al. observed 958 non-hospitalized patients with COVID-19 after 4 and 7 months from the acute phase. A lower baseline anti-SARS-CoV-2 IgG level, anosmia, and diarrhea during acute SARS-CoV-2 infection were associated with a higher risk of developing long-lasting signs and symptoms (17). Prolonged signs and symptoms had a significant impact on the quality of life. In the meta-analysis of 12 studies with 4,828 patients with post-acute COVID-19, the pooled prevalence of poor quality of life was 59% (95% CI 42%−75%) (18). Patients reported pain/discomfort (41.5%), anxiety/depression (37.5%), and difficulty with mobility (36%) or with usual activities (28%). The results of the research show the scale of the problem and the challenge of caring for patients with Long-COVID-19.

Important differences in the course of COVID-19 were already observed. The epidemiological data indicate that men experience more severe signs and symptoms and suffer higher mortality from COVID-19 than women (19, 20). Scully et al. showed that the average male COVID-19 fatal rate was 1.7 times greater than that of the female fatal rate in 37 European countries (21). The causes of this phenomenon are genetic factors and sex hormones that influence immune system regulation (22, 23). However, in our study, women more often reported moderate to severe signs and symptoms of COVID-19, which accounted for almost 70% of signs and symptoms in both groups. Due to the higher male mortality and the higher risk of severe courses, including hospitalization, women were more likely to be home-isolated patients. It should also be underlined that our definition of the COVID-19 course severity was based partly on subjective symptom reports. We observed that women have a 40% higher risk of Long-COVID-19. Some immunological differences such as the lower production of pro-inflammatory interleukin-6 (IL-6) after viral infection in women (24), the potentially protective role of estrogen and progesterone (25), and the higher expression of angiotensin-converting enzyme-2 (ACE2) and transmembrane protease serine 2 (TMPRSS2) receptors in men and women (24) could explain the higher occurrence of post-COVID-19 signs and symptoms. Moreover, according to research, higher levels of depression, poor sleep quality, and the presence of anxiety are more vulnerable in women, promoting Long-COVID-19 (26, 27). Similarly, in the course of cardiovascular diseases, the female sex is associated with lower mortality and also with a worse quality of life (28). This problem was also observed in our study group and an assessment of the life quality, following COVID-19, should be the subject of further analysis.

Many studies show that obesity and impaired metabolic health contribute to impaired immune responses (29–31). Phung et al. performed a meta-analysis of obesity and influenza-related pneumonia (32). They found that the risk of pneumonia among individuals with obesity (BMI ≥30 kg/m^2^) was increased by 1.33 times (95% CI 1.05–1.63) and 4.6 times (95% CI 2.2–9.8) among patients with morbid obesity (BMI ≥40 kg/m^2^). Such often association was observed among patients with COVID-19 (33). Obesity promotes a severe course of SARS-CoV-2 infection and increases the risk of respiratory failure (34–37). In our study, we have also observed an association between higher values of BMI and a symptomatic course of COVID-19. Furthermore, it seems that obesity-related chronic inflammations and processes of immunometabolism not only promote a severe clinical course of acute SARS-CoV-2 infection but also contribute to a Long-COVID-19 syndrome (38). However, we did not confirm this in our study group. Obesity and metabolic disorders as modifiable risk factors should be a subject of concern in patients during acute infections and follow-up examinations.

In connection with obesity as a risk factor for a severe course of SARS-CoV-2 infection, low-grade systemic inflammations are associated with the development of insulin resistance, dyslipidaemia, atherosclerosis, type 2 diabetes, hypertension, and asthma (39), i.e., comorbidities adversely affect the outcomes of patients with COVID-19 (11, 40). Meta-analyses of many studies demonstrated that arterial hypertension is the most common comorbidity that correlates with a severe course of COVID-19 (41, 42). Moreover, according to the same meta-analyses, diabetes was more prevalent among fatal cases and, likewise, respiratory diseases (41). Chronic diseases may also have an impact on the occurrence of Long-COVID-19; but according to research results, the relationship between cardiometabolic diseases and Long-COVID-19 is not clear as in the case of COVID-19 alone (43). Halpin et al. found that a pre-existing respiratory disease; a higher BMI; an older age; Black, Asian, and Minority Ethnic (BAME); and dyspnoea at the 4th−8th week of follow-up are associated with prolonged COVID-19 signs and symptoms (44). A pre-existing asthma is significantly associated with Long-COVID-19 (14).

In the course of many chronic diseases, lifestyle risk factors are associated with morbidity, mortality, and the loss of disease-free years of life (45–47). According to the results of our study, stress and overworking before the infection, and sleeping disturbances are associated with the course of COVID-19. Hamer et al. demonstrated, in a large-scale general population study, a dose-dependent association between the risk of COVID-19 and worsening lifestyle scores. The following factors, i.e., physical inactivity (OR 1.32, 95% CI 1.10–1.58), smoking (OR 1.42, 95% CI 1.12–1.79), and obesity (OR 2.05, 95% CI 1.68–2.49) had a higher risk (48). In the results of the analyses of hospitalized patients in Iran, approximately 82% of patients had insufficient physical activity, and 67.3% of patients were reported to have an unfavorable nutritional status. There was also a significant correlation between ICU admissions and unhealthy lifestyles (OR 0.40, *P* = 0.015) (49). The results of studies demonstrate an association between physical activity behaviors and viral defense responses (11, 50). Li et al. showed in a Mendelian study with randomization that BMI and smoking increase and physical activity might decrease the risk of severe course of COVID-19 (13). However, Rowlands et al. found among 2,009 patients with COVID-19 that the physical activity level was not significantly associated with the risk of testing positive for SARS-CoV-2 or of developing severe COVID-19. Furthermore, a worse balance between activity and sleep/rest with irregular sleeping hours was predictive (11). In contrast, prolonged stress impairs the immune system (51). In the meta-analysis of 23 studies, the presence of any mental disorder was associated with an increased risk of COVID-19 mortality (OR 2.00, 95% CI 1.58–2.54) (52). A small number of studies on the impact of lifestyle parameters on the occurrence of Long-COVID-19 have been performed so far. Although we did not find in our study a direct correlation between lifestyle parameters and the occurrence of prolonged signs and symptoms, indirectly through their impact on the severity of signs and symptoms of acute infections, which are a predictor of Long-COVID-19, one can suppose that such a relationship exists.

### Limitations

Besides the design of the study that precluded any consideration of causality, the present analysis has several other limitations. First, our study participants were not representative of all patients with COVID-19 as we excluded from the analysis all participants hospitalized for COVID-19. In contrast, our data provide a unique possibility to assess the factors related to the course of COVID-19 in non-hospitalized patients. The reliability of the data gathered relies on the credibility of the information provided by the study participants. Conversely, an important advantage of our analysis is that our results are not only based on abstracted medical record data but also involved face-to-face interviews and examinations using the same protocol and standardized methods and instruments. We could not analyse vaccination status. In addition, we were not able to identify the variants of SARS-CoV-2. The lack of evaluation of laboratory results should also be mentioned as a methodological limitation. Previous studies have shown that higher eosinophilia, higher neutrophil-to-lymphocyte ratios, lower platelet counts, higher serum ferritin levels, and higher serum bilirubin levels are associated with a higher risk of severe COVID-19 (53, 54). Finally, we could not analyse the data on COVID-19 treatment in the acute phase of the disease.

In summary, despite the limitations of our study, it provides reliable information on risk factors for the development of Long-COVID, including selected aspects of lifestyle, disease course, and chronic conditions. However, the present topic calls for further knowledge in this area. Therefore, further observations on representative groups based on standardized tools also assessing vaccination status and COVID-19 treatment are essential.

## Conclusion

Among non-hospitalized patients with confirmed SARS-CoV-2 infection, age, female sex, BMI, asthma, hypertension, stress or overworking, and night shifts are significantly related to the severity of the acute phase of the COVID-19, while female sex, asthma, history of myocardial infarction, and the severity of symptoms in the acute phase of COVID-19 are the predictors of Long-COVID. We did not find any independent relation between Long-COVID and the studied lifestyle factors.

## Data availability statement

The original contributions presented in the study are included in the article/supplementary materials, further inquiries can be directed to the corresponding author/s.

## Ethics statement

The studies involving human participants were reviewed and approved by Bioethics Committee of Lodz Regional Medical Chamber—No. 0115/2021. The patients/participants provided their written informed consent to participate in this study.

## Author contributions

Conceptualization: MC and MP-J. Data curation and methodology: MC, MP-J, and PJ. Formal analysis: MB and JK. Investigation, visualization, and writing—review and editing: MC, MP-J, MB, JK, and PJ. Project administration: MC. Supervision and validation: MC and PJ. All authors have read and agreed to the published version of the manuscript.

## Conflict of interest

The authors declare that the research was conducted in the absence of any commercial or financial relationships that could be construed as a potential conflict of interest.

## Publisher's note

All claims expressed in this article are solely those of the authors and do not necessarily represent those of their affiliated organizations, or those of the publisher, the editors and the reviewers. Any product that may be evaluated in this article, or claim that may be made by its manufacturer, is not guaranteed or endorsed by the publisher.
